# Comparison between methods of assessing lumbosacral curve obtained by radiographic image

**DOI:** 10.1590/1413-78522015230200840

**Published:** 2015

**Authors:** Daiane Aparecida Vacari, Eduardo Borba Neves, Leandra Ulbricht

**Affiliations:** 1 Universidade Tecnológica Federal do Paraná, Curitiba, PR, Brazil, 1. Universidade Tecnológica Federal do Paraná (UTFPR), Campus Curitiba, Curitiba, PR, Brazil

**Keywords:** Spine, Lumbosacral region, Radiography, Evaluation

## Abstract

**OBJECTIVE:**

To investigate the correlation between different radiographic methods in the evaluation of the lumbosacral concavity.

**METHODS:**

The sample consisted of 52 individuals with ages ranging from 18 to 28 years old. The procedures related to radiographic image collection were carried out in collaboration with a diagnostic imaging center of a hospital in Curitiba, PR, Brazil. The angles of the lumbosacral concavity were evaluated by the following methods: Centroid, Cobb1_L1-S1_, Cobb2_L1-L5_, Cobb3_L2-S1 _Cobb4_T12-S1, _Posterior Tangent and Trall.

**RESULTS:**

High correlation coefficients (r ranging from 0.77 to 0.89) were found among variations of the Cobb method. Additionally, we propose a categorical classification of angle values obtained by each method. We also analyzed the influence of the level of the inflection point between the lumbar lordosis and thoracic kyphosis in determining the evaluation method to be used. The inflection point had a higher incidence in the region between the twelfth thoracic vertebra and the first lumbar vertebra (63.5%).

**CONCLUSION:**

The correlation and agreement between methods vary considerably. Moreover, the thoracolumbar inflection point should be considered when choosing the method of assessing patients.

**Level of Evidence I, Diagnostic Study.:**

## INTRODUCTION

Formed by a complex anatomical structure, the lumbosacral spine is a very researched segment in the scientific midst.^1^^,^ 2 The demands arising from the increasing number of cases related to back pain and affections in the lumbosacral structure are usually from poor posture, and in most cases arising from the lack of adaptation of work positions in labor activities, inactivity or congenital and chronic illnesses^3^. In order to prevent the disorders of the lumbosacral spine, the pattern of postural alignment considered optimal should have characteristics of bone muscle, ligament, tendon and joint uniformity, and besides, it should present the proper functioning of the nervous and labyrinthic system.^2^

Due to the constant demands of scientific knowledge related to the spine, many techniques have been developed to analyze and diagnose cases of posture abnormalities. The currently most efficient are X-ray and Magnetic Resonance Imaging.^4^

On the evolution of capture techniques, we observe the progress of the methods aimed at measuring radiographic images.^5^ In order to be reliable, diagnosis regarding this type of image ought to use methods with high levels of inter-rater reliability. Currently, tests like Cobb, Trall, Centroid and Posterior Tangent have such characteristics that demonstrate correlatable results, as showed by the study of Lee *et al*.^6^

However, statistical investigations between the various existing methods for the measurement of lumbosacral curvature remain restricted. Statistical inference addressed in most studies is related to the investigation of reliability intra and interevaluaters.^6^ Therefore, the motivation of this study was to correlate and verify the agreement between the following methods: Cobb, Centroid, Trall, and Posterior Tangent and to propose a categorical classification of the methods studied. 

## MATERIALS AND METHODS

The methodology for the design of this study was observational, quantitative descriptive and transversal. In total, 58 individuals volunteered to collection, male and female, aged 18-28 years old. Regarding inclusive requirements for the selection, it was determined that participants would fit between the ages 18 to 35 years old, present a morphological profile compatible with the result of the waist/hip ratio (WHR) ranked from low to moderate, had undergone an assessment by an orthopedic doctor for indication of radiograph exam and delivered the Free and informed consent form duly signed.

Individuals who had undergone some type of radiographic examination in the last year, participants who were not selected by the evaluation of the orthopedic doctor and women, specifically, who were pregnant or nursing were not included in the data collection. Due to the exclusionary factors, the final sample included 52 individuals.

The procedures of radiographic collection were carried out in partnership with a diagnostic imaging center of a hospital in the capital of Curitiba, PR, Brazil. Radiographies of the lumbosacral spine were performed by a radiology professional, at the profile (P) and anteroposterior (AP) incidence and stored in digital format for further analysis. As investigative criteria of this research, we used only images taken in profile, and other images captured in AP were used for diagnosis of orthopedic surgeon applicant.

This research project was submitted to the Research Ethics Committee of *Centro Universitário Campos de Andrade* and was approved under protocol Nº438.

In the radiographic image capture process the equipment used were: a Buck Mural Philips Optimus emitter, a Philips digital control desk, a Regius radiographic film and Computer Radiography (CR) system and the Konica Minolta manager software, which enabled the editing of images taken by CR. Radiographic exams were performed in the afternoon, always by the same professional. The postures adopted by the participants were standardized according to the obtained incidences. 

The 1.46r ImageJ software was used for determining the lumbosacral angle according to the following methods: the four variations Cobb (Cobb_L1-S1_; Cobb_L1-L5_; Cobb_L2-S1 _e Cobb_T12-S1_); centroide; Trall; and posterior tangent.

Statistical Analysis

The sample was characterized by descriptive statistical treatment, with the following indicators: minimum value, maximum value, mean, standard deviation and sample size. The inferential statistical analysis started from the Kolmogorov-Smirnov normality test to verify the normality of the distribution of the study variables, with significance level 95% (p <0.05).^7^

Once the data were tabulated, the Pearson linear correlation coefficients between the studied protocols were calculated. After that, each evaluation protocol of the lumbosacral angle was stratified into five categorical tracks, from the value of the means and standard deviations found. (Table 1)

**Table 1.** Classification of angular values.

Interval	Category
< (M – 2SD)	Far below
(M – 2SD) a (M – 1SD)	Moderately below
(M – 1SD) a (M + 1SD)	Average
(M + 1SD) a (M + 2SD)	Moderately above
> (M + 2SD)	Far above

M: Mean; SD: Standard deviation.

Then the agreement intensities between the classifications of the protocols studied were evaluated using the Kappa agreement coefficient.^8^

## RESULTS

The sample consisted of 52 individuals, of which 65.4% (n=34) corresponded to male subjects and 34.6% (n=18) to female. The variables related to body weight and height corresponded to the mean and standard deviation of 68.9±10.1 kg and 172.9±8.9 cm and the mean age was 20.7±2.6 years old.

From the sample group chosen for the study, the Pearson correlation test was applied to all methods employed. Table 2 shows the correlation coefficient, its statistical significance and the correlation of the intensity rating for all methods.

**Table 2.** Pearson correlation coefficients between radiographic methods.

	Cobb_2 L1s_L5i_	Cobb_3 L2s_S1s_	Cobb_4 T12i_S1s_	Centroide	Posterior _L1xL5_	Posterior _L1xS1_	Trall
Cobb_1 L1s_S1s_	0.809**	0.810**	0.893**	0.611**	0.467**	0.548**	0.693**
	Strong	Strong	Strong	Moderate	Moderate	Moderate	Moderate
Cobb_2 L1s_L5i_		0.800**	0.820**	0.664**	0.502**	0.603**	0.707**
		Strong	Strong	Moderate	Moderate	Moderate	Strong
Cobb_3 L2s_S1s_			0.765**	0.572**	0.444**	0.627**	0.677**
			Strong	Moderate	Moderate	Moderate	Moderate
Cobb_4 T12i_S1s_				0.585**	0.451**	0.618**	0.651**
				Moderate	Moderate	Moderate	Moderate
Centroid					0.493**	0.276*	0.697**
					Moderate	Weak	Moderate
Posterior _L1xL5_						0.631**	0.474**
						Moderate	Moderate
Posterior _L1xS1_							0.360**
							Weak

* p<0.01.

In addition to the linear correlation test the angle values found in each method were classified into five categories: far below; moderately below; average; moderately above and far above. The limits of each categorical group were established from the mean values and standard deviations obtained for each method. (Table 3)

**Table 3.** Categorical classification of angular values according to lumbar curvature identification method

Classification	Cobb_3L2s - S1s_	Cobb_2L1s - L5i_	Cobb_1L1s - S1s_	Cobb_4T12i - S1s_	Centroid	Posterior _L1-L5_	Posterior _L1-S1_	Trall
Mean	50.38	41.98	55.41	58.14	35.54	29.33	60.52	35.14
Standard deviation	9.83	10.80	10.68	9.59	11.20	9.54	11.64	7.54
Far below	< 30.72	< 20.38	< 34.05	< 38.96	< 13.13	< 10.24	< 37.24	< 20.06
Moderately below	30.72 to 40.55	20.38 to 31.18	34.05 to 44.73	38.96 to 48.55	13.13 to 24.34	10.24 to 19.78	37.24 to 48.88	20.06 to 27.60
Average	40.56 to 60.20	31.19 to 52.78	44.74 to 66.08	48.56 to 67.74	24.35 to 46.74	19.79 to 38.87	48.89 to 72.16	27.61 to 42.68
Moderately above	60.21 to 70.03	52.79 to 63.58	66.09 to 76.76	67.75 to 77.33	46.75 to 57.95	38.88 to 48.42	72.17 to 83.80	42.69 to 50.22
Far above	> 70.03	> 63.58	> 76.76	> 77.33	> 57.95	> 48.42	> 83.80	> 50.22

The interpretation proposed by Landis and Koch,^8^ to categorize Kappa statistic values, whose values and agreement intensities are shown in Table 4.

**Table 4.** Kappa statistics between lumbar curvature methods.

	Cobb_1L1 - S1_	Cobb_2L1 - L5_	Cobb_3L2 - S1_	Cobb_4T12 - S1_	Centroid	Posterior _L1-L5_	Posterior _L1-S1_	Trall
Cobb_1_		0.634	0.351	0.491	0.322	0.052	0.366	0.215
		Strong	Acceptable	Moderate	Acceptable	Slight	Acceptable	Acceptable
Cobb_2_			0.401	0.545	0.503	0.113	0.344	0.315
			Acceptable	Moderate	Acceptable	Slight	Acceptable	Acceptable
Cobb_3_				0.345	0.231	0.198	0.427	0.247
				Acceptable	Acceptable	Slight	Moderate	Acceptable
Cobb_4_					0.260	0.159	0.395	0.240
					Acceptable	Slight	Acceptable	Acceptable
Centroid						0.001	0.140	0.241
						Slight	Slight	Acceptable
Posterior _L1-L5_							0.241	0.452
							Acceptable	Moderate
Posterior _L1-S1_								0.033
								Slight

The ratings of the angles from Cobb1 and Cobb2 methods showed strong agreement and the highest Kappa index - 0.634 - between all methods.

Besides the proposed categorization process for methods of measurement of lumbar curvature, we identified the vertebrae that presented a higher frequency on the inflection point.

**Figure 1.**
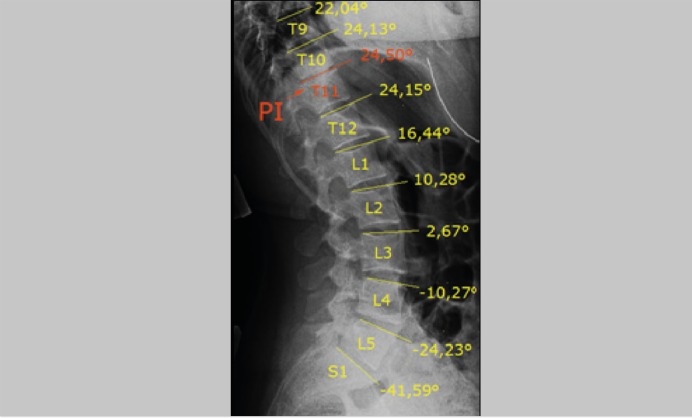
Procedure for detecting the inflexion point of the thoracolumbar spine.

For measuring such a procedur it required the Straight tool and ImageJ software for the design of the upper end plate of the last thoracic vertebra and first lumbar vertebra. After determining each of these structures, the results of the slopes of each vertebral body were compared and, from the moment of results inversion, i.e. after the angular values are no longer increasing or decreasing and reverse at a given vertebra , the inflection point (IP) was located, as evidenced in Figure 1.

It can be observed in the radiographic image (Figure 1) that the identification of the inflection point is very discreet, making it difficult to perform the procedure at the clinic. For the vertebrae T9, T10 and T11, low frequency percentage of the inflection point were found. (Table 5)

**Table 5.** Descriptive statistics of the inflexion points on the thoracolumbar curvature.

Vertebra	%	N
T9	1.9	1
T10	3.9	2
T11	9.6	5
T12	38.5	20
L1	25.0	13
L2	21.1	11
Total	100.0	52

## DISCUSSION

**Figure 2.**
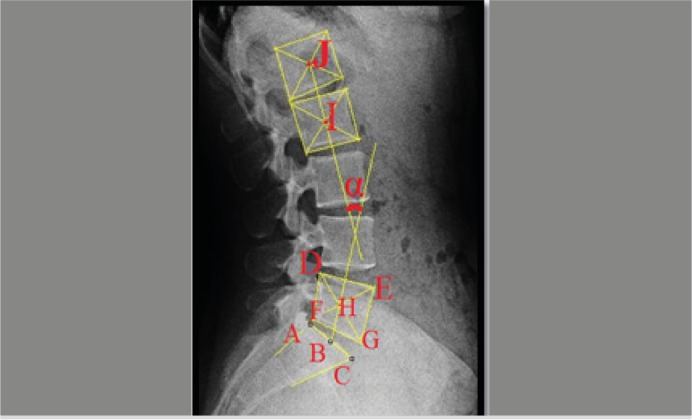
Procedure for performing the centroid method.

The results show high correlation coefficients among variations in Cobb method: Cobb_4 T12i-S1s_ e Cobb_1L1s-S1s_ (0,893), Cobb_4 T12i-S1s_ e Cobb_2 L1s-L5i_ (0,820), Cobb_3 L2s-S1s_ and Cobb_1 L1s-S1s_ (0,810). In these methods, firstly, two dividing lines parallel to the surface of the vertebral bodies are marked, selected in each method and subsequently crossed by two perpendicullar lines, which result in the angles, as shown.^5^^,^ 6 ^,^ 9 ^-^ 12 (Figure 2)

Despite being widely used in clinics for diagnosis of spine pathologies, we did not identify in the literature review studies that compared all four methods. However, some studies prioritized comparing the reliability of the methods Cobb _L1-S1_ and Cobb _L1-L5_, the results indicated interclass correlation coefficients (ICC) of 0.96 and 0.97, for Cobb _L1-S1_ and Cobb _L1-L5_ intraobserver and values of 0.95 and 0.96 for Cobb _L1-S1_ and Cobb _L1-L5_ interobservers, respectively. These facts corroborate the strong correlative evidence also found in this study.^6^ The purpose of comparing those Cobb methods emerges from the failure in obtaining radiographic image, because defects such as blurring, granulation, dimming or overlaping organs in the areas studied may compromise the identification of anatomical reference points of the selected test, thus hindering to obtain the correct measurement point. 

**Figure 3.**
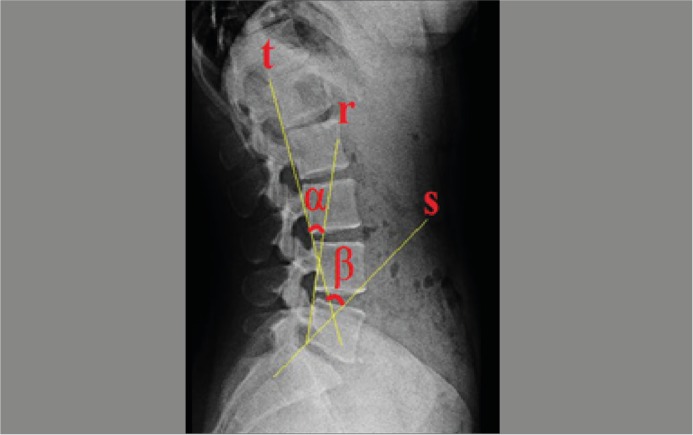
Procedure for performing the posterior tangent method.

The centroid method, also used in this study, allows making marks that follow the actual contour of the vertebral bodies, therefore, decreasing the negative influence caused by the irregularity of the vertebrae in angular measurements.^5^ (Figure 3) From this theory, Chen^13^ compared the centroid method, with two tests: Cobb_L1-L5_ and Cobb_L1-S1_. The results of inter and intraobservation with three different evaluators showed that, among all, the centroid method had the highest average interclass correlation, Cobb_L1-L5_ (0.82), Cobb_L1-S1_ (0.78) and centroid (0.90 ). Besides the correlative approach of the centroid method compared to other tests present in this sample, it was possible to identify through categorization, the reference angle values for a population sample of the Southern region of Brazil. This contribution defines a framework of angle values, considering the absence of such data in the current literature.

**Figure 4.**
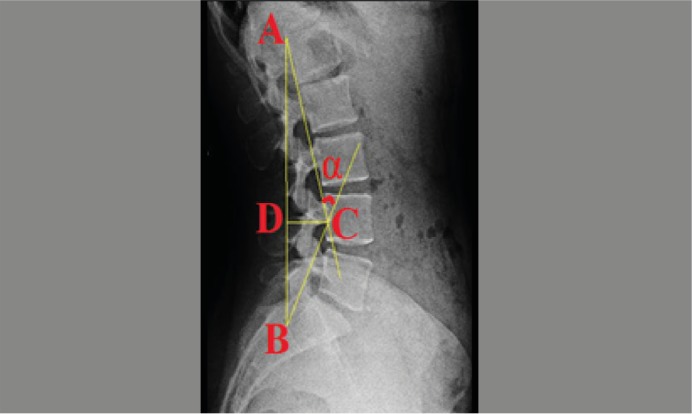
Procedure for performing the Trall method.

The posterior tangent method (Figure 4) uses a traced line that demarcates the extension of the posterior wall of the vertebral body of L1 and another indicating S1. The intersection of the two lines derived from later portions of the aforemenined vertebrae generate the angular measure. Lee *et al*.^6 ^found that the measurement of the posterior tangent angle is more reproducible, because it showed the highest rate of reliability in comparisons to Cobb_L1-S1_. In this study, there was a moderate correlation (r = 0.63) between these two methods. The moderate result can be explained by the fact that both methods use the same anatomical points, L1-S1. Despite the geometric patterns are different, it is considered as a priority intervening the positioning of the vertebral body, as described in Chen's work.^13^

**Figure 5.**
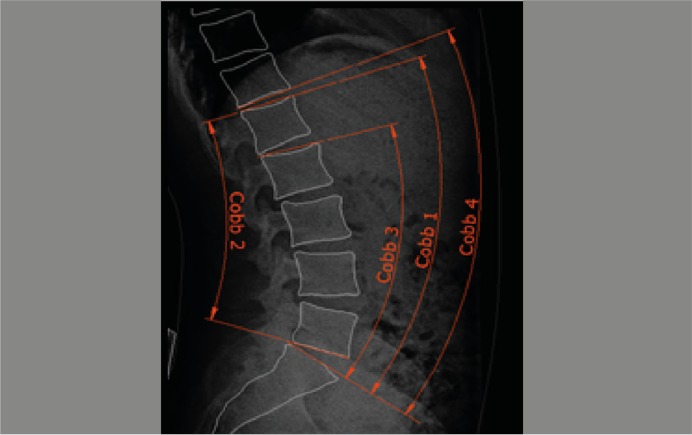
Trace of angles Cobb1 L1-S1, Cobb2 L1-L5, Cobb3 L2-S1 and Cobb4 T12-S

The Trall method was designed from the intersection of straight lines that run from posterosuperior vertex L1 vertebra and the posterior-vertex of S1 vertebra, crossing over the point of greatest depth of lordosis, ie, the point of greatest distance from the line passing on the vertices. (Figure 5) The origin of this measure came in order to override the method of variations, so existing, Cobb. In addition, it presents greater reproducibility, with the design of differential curvature of the lumbar spine and suffers less variations of the vertebral bodies in its contradiction medida.6,14,15 in this study there was a strong correlation (r = 0.70 ) between the method of Trall and the methodology of Cobb2 L1s-L5i. This result can be explained by the influence of the L1 vertebra Furthermore, comparisons with other methods showed moderate Cobb correlations.

As for categorical classification it was not possible to identify the reference values indicated for each method studied, except for the Cobb_L1-L5_ method, whose reference values range from 40 to 60 degrees.^16^ From this fact, it was noted the necessity for a categorization for fundamented assessment to the other methods.

The relations obtained from the Kappa agreement results showed correlative indexes of 0.63 for the angles Cobb_1_ and Cobb_2_ between moderate measures. Cobb_4_ method when compared to Cobb_1_ and Cobb_2_ reached indices of 0.49 and 0.54, respectively. These findings show that the measurements performed with the same angular pattern show proven stronger relations.^13 ^Possibly those results rated as reasonable and weak indicate the discrepancy between the measurement points, and analyzing this reason the identification of the inflection point is discussed.

Then, it has been shown that in the current sample, the vertebra occurring in the inflection point which appeared more frequently was T12, with 38.5% (n=20). In the case of vertebrae L1 and L2, the occurrence of the inflection point occurred at 25.0% (n=13) and 21.1% (n=11) of patients, respectively.

The identification of the inflection point of the thoracolumbar spine, and its influences are not widely used in the present study, which focused the structure of the backbone, although there are investigative work, as proposed by Singer *et al*.,^17^ who reported the presence of the inflection point between vertebrae T10 and T12 in 286 sagittal X-rays. Other evidences regarding studies that have suggested that, besides determining the inflection point in the coordinate system of the spine showed that, in addition to determining more accurately the start of the lumbar curvature, this point may help the quantitative analysis of the lumbar curvature in two planes simultaneously (coronal and sagittal).^18^

For example, to the vertebrae above T12, the angular Trall test should be applied. As for the inflection points found between T12 and L1, the methods Cobb_1 L1s-S1S_; Cobb_2 L1s-L5i_; and posterior tangent_L1-L5_ can be recommended for demarcation. For the identification of the inflection point from the second lumbar vertebra the, Cobb_3 L2s-L1s_ methods are indicated. Finally, in all participants presenting the inflection point below the L2 vertebra the centroid method is recommended for viabilizing measurement at any point of inflection.

To verify the effectiveness of the influence of the inflection point, there was a correlation between the techniques indicated in the same inflection location. As for vertebrae T12 and L1 more than one technique was indicated, which showed a moderate to strong correlation between them.

Thus, Table 6 shows a suggestion of which methods are the most appropriate for each individual, in accordance with the inflection point region.

**Table 6.** Classification defined to division of anatomical regions and respective recommended methods.

Vertebra classification	Indicated methods	Percentage of the sample
Above T12	Trall	15.4%
T12 – L1	Cobb_4 T12i-S1s_	63.5%
	Cobb_1 L1s-S1s_	
	Cobb_2 L1s-L5i_	
	Posterior _L1-L5_	
L2	Cobb_3 L2s-S1s_	21.1%
Below L2	Centroid	0.0%
Total		100.0%

## CONCLUSION

It was possible to identified with this study that the correlative and agreement values were most significant for the Cobb_1 L1-S1_, Cobb_2 L1-L5_, Cobb_3 L2-S1 _and Cobb_4 T12-S1_ methodology. However, we also observed a strong correlation (r = 0.70) between Cobb_2_ and Trall methods. Therefore, it can be concluded that the diversity of anatomical references used in the studied techniques should be considered when choosing a techique to a given patient, depending on the placement of the thoracolumbar inflection point, since knowledge of its location allows the choice of technique to be used more accurately.
